# The default network is causally linked to creative thinking

**DOI:** 10.1038/s41380-021-01403-8

**Published:** 2022-01-01

**Authors:** Ben Shofty, Tal Gonen, Eyal Bergmann, Naama Mayseless, Akiva Korn, Simone Shamay-Tsoory, Rachel Grossman, Itamar Jalon, Itamar Kahn, Zvi Ram

**Affiliations:** 1 Department of Neurosurgery, Tel Aviv Sourasky Medical Center and The Sackler Faculty of Medicine, Tel Aviv University, Tel Aviv, Israel; 2 Functional Brain Center, Wohl Institute for Advanced Imaging, Tel Aviv Sourasky Medical Center, and The Sagol Center for Neuroscience, Tel Aviv University, Tel Aviv, Israel; 3 Department of Neuroscience, Rappaport Faculty of Medicine and Institute, Technion—Israel Institute of Technology, Haifa, Israel; 4 Department of Psychology, University of Haifa, Haifa, Israel; 5 School of Medicine, Stanford University, Stanford, CA USA; 6 Department of Psychology, Tel Aviv University, Tel Aviv, Israel; 7 Present Address: Department of Neuroscience, Zuckerman Mind Brain Behavior Institute, Columbia University, New York, NY 10027 USA

**Keywords:** Neuroscience, Biological techniques

## Abstract

Creative thinking represents a major evolutionary mechanism that greatly contributed to the rapid advancement of the human species. The ability to produce novel and useful ideas, or original thinking, is thought to correlate well with unexpected, synchronous activation of several large-scale, dispersed cortical networks, such as the default network (DN). Despite a vast amount of correlative evidence, a causal link between default network and creativity has yet to be demonstrated. Surgeries for resection of brain tumors that lie in proximity to speech related areas are performed while the patient is awake to map the exposed cortical surface for language functions. Such operations provide a unique opportunity to explore human behavior while disrupting a focal cortical area via focal electrical stimulation. We used a novel paradigm of individualized direct cortical stimulation to examine the association between creative thinking and the DN. Preoperative resting-state fMRI was used to map the DN in individual patients. A cortical area identified as a DN node (study) or outside the DN (controls) was stimulated while the participants performed an alternate-uses-task (AUT). This task measures divergent thinking through the number and originality of different uses provided for an everyday object. Baseline AUT performance in the operating room was positively correlated with DN integrity. Direct cortical stimulation at the DN node resulted in decreased ability to produce alternate uses, but not in the originality of uses produced. Stimulation in areas that when used as network seed regions produced a network similar to the canonical DN was associated with reduction of creative fluency. Stimulation of areas that did not produce a default-like network (controls) did not alter creative thinking. This is the first study to causally link the DN and creative thinking.

## Introduction

Creativity, traditionally described as the ability to produce novel and useful ideas [1], has played a major role in the establishment of modern culture and civilization. Creative thinking is essential to the daily functioning of modern human beings and allows for inspirational problem solving, technological and artistic advancement. This complex, multi-factorial cognitive process cannot be linked to a specific cerebral anatomical area, such as primary motor or sensory cortices, or to the activity of individual neurons, and instead is assumed to be network-dependent [2]. With the development of robust whole-brain functional imaging, higher cognitive functions are increasingly associated with large-scale cortical networks. These networks are comprised of distributed neuronal ensembles that are spatially dispersed but temporally synchronized [3]. Large-scale neuronal networks are nowadays decipherable using advanced functional imaging and computational methods and are thought to subserve complex abilities, such as attention, executive function, and more [4–6]. The Default Network (DN) is a well-explored, large-scale, association cortical network traditionally linked with internal mentation [7, 8]. Its increased activity is associated with decreased performance in externally driven tasks, and it conversely demonstrates task-induced deactivation when focus is shifted to external stimuli [9]. Recent evidence suggests that connectivity between areas traditionally associated with the DN may underlie the ability to carry out creative thinking [1, 10]. In addition, DN interactions with the salience and executive networks, ensembles that are not traditionally functionally linked, are thought to underlie creative thinking [11].

Heretofore, despite many studies demonstrating correlation between the DN, other large-scale cortical networks, and creativity, no causal relation has been shown. While there have been publications describing transcranial disruption of an anatomical area, and associated impairment in DN related functions such as episodic memory [12] and simulation [13], these have targeted the canonical DN and did not use individualized DN mapping. In addition, several technical limitations associated with transcranial stimulation limit the ability to establish such causality or identify relationships at the individual participant level.

**Fig. 1:**
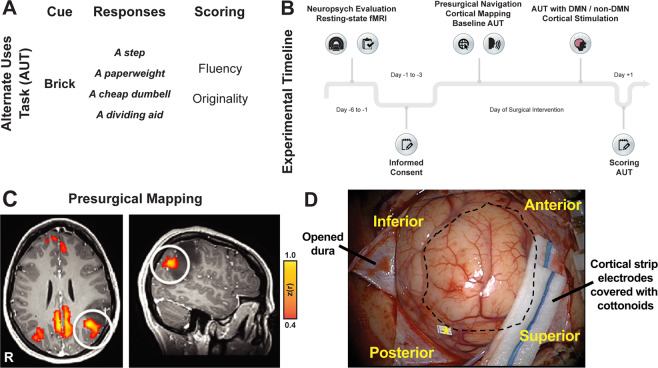
Study design. **A** Alternate uses task (AUT). An example of a task cue, followed by potential common alternate uses a participant may produce. Each item was scored for fluency and originality creativity sub-components. **B** The experimental timeline from participant recruitment to postoperative period. **C** Individualized default network (DN) map. Seed-based whole-brain correlation maps were derived from the posterior cingulate cortex (PCC) region of interest. For each study participant, similar DN functional connectivity was derived individually and a target stimulation site was classified either as DN node (white circle) or non-DN site (i.e., outside the correlation map; not shown here). Stimulation sites were chosen such that they would be accessible intraoperatively. **D** An example intraoperative view of exposed tumor (dotted black line represents tumor surface circumference) and the target DN node for direct cortical stimulation as identified by intraoperative navigation (yellow asterisk).

We utilized the unique experimental platform of awake brain surgery for resection of a primary brain tumor to explore the effects of DN node stimulation. During routine surgery near eloquent brain regions, cortical mapping via electrical stimulation (direct cortical stimulation, DCS) is performed to identify functional cortical areas and thus preserve crucial functions, such as speech. Traditionally, intraoperative mapping identifies basic functions of sensation, motor, and speech; higher cognitive functions that are network-dependent are not mapped. DCS allows for the temporary inhibition of a limited cortical area using a bipolar stimulator that minimizes current spread to a ~2 mm sphere [14]. To investigate the possible connection between the DN and creative thinking, we examined the effect of DCS of a preoperatively-identified DN node on the ability to perform creative thinking. Patients who agreed to participate in this study underwent DCS of an individually mapped DN node (study) as extracted from preoperative resting state-based functional connectivity fMRI mapping of this network, or a non-DN stimulation (control). To evaluate creative thinking, we utilized the *alternate uses task* (AUT; Fig. 1A), a well-validated creativity task in which the subject needs to provide varied, alternate possible uses for everyday common objects [15]. The AUT assesses divergent thinking, which is widely considered to be an important antecedent of creativity because it involves the ability to consciously generate new ideas that branch out and allow for many possible solutions to a given problem, and is scored based on the originality and the number of valid uses [15]. The effects of stimulation on two main components of creative thinking, originality and creative fluency, as expressed by the relative domains in the AUT, were examined. We hypothesized that disruption of DN synchronization by artificial non-physiologic electrical stimulation would affect the ability to perform creative thinking (Fig. 1B).

## Methods

### Patient selection and experimental procedure

All patients participating in this experiment willfully signed an informed consent form, and this experiment was approved by the Tel Aviv Medical Center ethics committee (0378-16-TLV). Included were patients with gliomas that were located in or near eloquent brain areas in the dominant hemisphere and were scheduled for clinically-indicated awake craniotomy for resection of their tumor. All patients had intact language functions preoperatively, and Hebrew was their native tongue. All patients underwent a thorough pre-operative neuropsychological evaluation, fMRI for determination of language dominance, and mapping of their DN (see below for details). Neurocognitive profiles were assessed preoperatively using the NeuroTrax computerized cognitive testing battery (NeuroTrax Corp., Bellaire, TX) [16]. Each patient’s DN map was uploaded to the intraoperative neuro-navigation system (BrainLab AG, Feldkirchen, Germany) and used clinically to locate the tumor and plan the craniotomy and subsequent resection of the tumor. After rigid positioning in a head holder in the operating room, each patient underwent a baseline evaluation of language functions. Next, a tumor-tailored craniotomy was done under regional nerve blocks and local anesthetics as is customary in our center [17, 18]. Mapping for language functions was carried out following craniotomy and dural opening, covering the entire exposed cortical surface. Intraoperative mapping was carried out in two stages. First, in order to control for interference with language functions, naming, verb generation, and comprehension were tested. Language function baselines were established inside the operating room following cortical exposure, but before cortical stimulation, to control for anxiety and drug related effects on performance and difference in experimental setting. If any language effects were noted during mapping, or if clinical/sub-clinical electrical seizures were identified (by intraoperative electrocortiography) during DCS the participant was excluded from this study. Then, a baseline AUT consisting of five items was performed with no stimulation. Next, we located the DN node as mapped preoperatively using the intraoperative navigation system and, when possible, stimulated the node while the participant performed the AUT. The final intraoperative location of the DCS was documented on the neuro-navigation system. For control participants who did not have a DN node close to the craniotomy, we stimulated a cortical area that was verified to be outside the DN as mapped preoperatively, and the stimulation site was documented. Each participant’s performance on the AUT was subsequently scored by a neuropsychologist (blind to the stimulation location) and results were compared to baseline and between study and control groups.

### DN mapping

Brain imaging was performed at the Wohl Institute for Advanced Imaging, Tel Aviv Medical Center, using a Siemens MAGNETOM Prisma 3.0 T scanner with a twenty-channel head coil. Anatomical 3D T1-weighted imaging was obtained using SPGR/FLASH sequences with 1 mm isotropic voxels. All functional whole-brain scans were performed with gradient echo-planar imaging (EPI) sequence of functional T2*-weighted images.

### Scan parameters, preprocessing and functional connectivity analysis

For each participant, a six-minute resting-state fMRI scan was acquired (TR = 3000 ms, TE = 35 ms, flip angle = 90°, 44 slices at 3 mm thickness with no gap, 117 repetitions). Preprocessing was done in agreement with widely available pipelines, and as previously described [19, 20]. A functional connectivity measure of the DN integrity was computed as the correlation between time courses in two DN ROIs, posterior cingulate cortex (PCC) and medial prefrontal cortex (mPFC). Specifically, spherical ROIs with 6 mm radius were defined with centers at: *x* = 0, *y* = −60, *z* = 30 for PCC and −1, 49, 5 for mPFC (coordinates defined in the Montreal Neurological Imaging [MNI] atlas space). Individual DN integrity was calculated as the whole brain connectivity of the PCC ROI (thresholded at *z*(*r*) of 0.4). Similarity between individual DN maps derived from a stimulation ROI and the canonical DN was estimated using the Sørensen-Dice similarity coefficient between the stimulation seed-based map (thresholded at *z*(*r*) of 0.2) and the DN from Yeo et al. [3].

### Alternate uses task (AUT)

Different items for the AUT were used at baseline and during stimulation to avoid learning effects and were identical across all participants (baseline—shoe, button, bedsheet, nail, paper clip; stimulation—box, car tire, cup, pencil, can). Participants were blinded to the study rationale and task and were informed that the study goals were to improve the ability to avoid resection-induced deficits on language and related high-order cognitive functions. Patients were only instructed to provide alternate uses for the sample item (newspaper, and its possible alternate uses – start a fire, wrap garbage, swat flies, fill boxes, line drawers, make up kidnap note, for example) before the baseline task was performed and again before the stimulation task. Following Shamay-Tsoory and colleagues [21, 22], participants were asked to think of original uses of everyday objects. Participants were given up to one minute to complete each item of the task for a total of 5 minutes for each part of the experiment. The intraoperative testing was recorded and documented (following participants’ consent) and blindly graded postoperatively. Measures of originality, based on a previously validated infrequency measure, and fluency, average number of valid creative uses given for each task item, were derived. For the fluency score, the number of alternative uses that did not recapitulate the original use (i.e., pencil—to write, to draw etc.) were counted. For the originality score, we utilized a previously constructed and validated answer database of 92 normal healthy adults. This allowed for a valid response frequency estimation. Each use was scored based on the infrequency in this answer database (score of 0 if >5% of participants provided it, 1 is 2–5%, 2 if <2%). For example—alternate uses for a shoe (common use to wear on feet) included “door stopper” 14.1%, score of 0 in originality, “make loud noises—bang with” 2.1% score of 1, and “put on the head for balance training” 1.08% score of 2. According to this, average originality and creative fluency scores were calculated for each patient and for each experimental condition. This scoring method is well validated and was chosen as it is objective, thus minimizing observer bias [23, 24]. In addition, this advantage is especially important as this study focused on the change in AUT score following DN stimulation, and did not attempt to predict future overall creative ability based on the baseline score. Both the participant and the examiner were blinded to the direct cortical stimulation location.

### Intraoperative direct cortical stimulation

Direct cortical stimulation was performed using the Ojemann cortical stimulator (Radionics Inc., Burlington, MA, USA). For language mapping, current intensity was modified by increasing the amplitude in 2 mA increments, from a baseline of 2 mA up to 8 mA. If seizures were observed, ice water was irrigated over the brain, and the study was aborted. During cortical mapping for language functions, observed behavioral effects and performance dysfunctions (e.g., speech arrest, anomia, etc.) were tagged and documented with regard to both anatomical and radiological location, as well as to the applied current intensity. If no effect was produced following language mapping, the stimulator was set to 4 mA, and stimulation was performed for 10 sec followed by 10 sec without stimulation until the completion of the task item.

## Results

A total of 13 out of 22 eligible patients who underwent awake resection of a primary brain tumor were included in this study (Table 1). Nine patients were excluded from this study, six due to intraoperative clinical or electrocorticographic seizures and 3 due to speech impairments induced during language mapping of the exposed cortex.

**Table 1:** Cohort demographics and AUT results.

#	Age	Gender	Tumor location	Pathology	Planned target stimulation site	Baseline originality	Baseline fluency	Stimulation originality	Stimulation fluency
1	24	F	Parietal	AA	DN	0.9	3	1.558333	2.085
2	26	M	Temporo-Insular	Astro	DN	0.48	3.6	0.81	2.88
3	60	M	Temporal	GBM	DN	0.66	1.6	0	1.5
4	69	M	Parietal	GBM	DN	0.83	3	0.418333	2.6
5	19	F	Frontal	Astro	DN	2	1	0.2	0.4
6	24	M	Temporal	Astro	DN	0.53	3.33	1	2.2
7	51	F	Insula	Oligo	DN	1.67	2	1.41	1.6
8	41	M	Frontal	Astro	DN	0.3	3.6	0.85	2.8
9	22	M	Temporo-Parietal	Astro	DN	0.5	2.667	0	1.5
10	34	M	Frontal	GBM	Control	0.4	1	0.625	2.125
11	32	M	Frontal	Oligo	Control	0.5	3.67	0.0975	1.1675
12	29	M	Temporal	Oligo	Control	0.43	3.75	0.35	4
13	20	M	Temporal	Astro	Control	0.55	2.33	1	0.5

*F* female, *M* male, *AA* Anaplastic Astrocytoma WHO grade III, *Astro* Astrocytoma WHO grade II, *GBM* Glioblastoma Multiforme WHO grade IV, *Oligo* Oligodendroglioma WHO grade II, *DN* default network.

All patients were high-functioning and cognitively intact (see table S1 for detailed cognitive assessment available for 12/13 patients) with no language disturbances at baseline following a preoperative neuropsychological evaluation. In addition, all patients were neurologically intact as documented in their hospital admission neurological examination. All patients had undergone assessment of a baseline creativity score of >0 in both AUT domains (originality and fluency of creative thinking), and thus were eligible to participate in this experiment. All patients willfully agreed to participate and signed an informed consent prior to surgery. Nine participants underwent stimulation of a DN node as derived from resting-state functional connectivity MRI posterior cingulate cortex (PCC) region of interest (ROI) seed-based whole-brain correlation map. Cases where the node was in proximity to the tumor, and inside the cortical region that was meant to be exposed according to a clinically-planned craniotomy participated in the experiment as subjects, and underwent stimulation of the node. Four participants who did not have an accessible DN node underwent stimulation of a non-DN cortical area and served as the control group. Importantly, prior to participation in this experiment, the exposed cortex was mapped intraoperatively for language functions as is the case with all awake craniotomies performed at our center. If any language disturbances were produced, or if a seizure (clinical or subclinical, as recorded by electrocorticography) was induced by the stimulation, the participant was excluded from this study (Fig. 1).

**Fig. 2:**
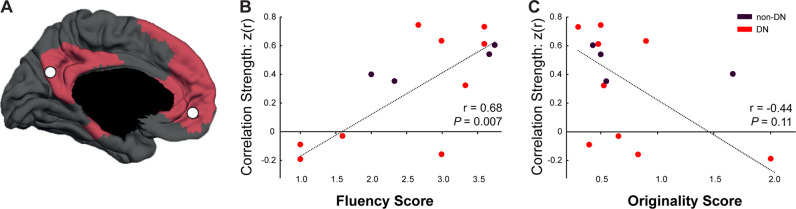
Intraoperative performance in the Alternate Uses Task (AUT) relative to Default Network (DN) resting-state functional connectivity MRI strength. **A** For each participant, the correlations between two key nodes of the DN, PCC and mPFC were computed. The extent of DN coverage, derived from Yeo et al. [3], is depicted on the medial surface (red) along with the PCC and mPFC seed region-of-interest locations (denoted by white filled circles). **B** AUT fluency score at baseline (no stimulation condition) is correlated with PCC–mPFC Fisher’s z-transformed Pearson’s *r* correlation strength (*z*(*r*)). **C** AUT originality score is not correlated with PCC–mPFC correlation strength.

To validate AUT in the operating room setting, we correlated the baseline scores obtained during surgery (prior to stimulation) with each individual participant’s correlation strength (z(r)) between PCC and the left medial prefrontal cortex (mPFC), a surrogate marker of DN integrity (*14*, Fig. 2A). Fluency was positively correlated with PCC-mPFC correlation (*r*_(11)_ = 0.68, *P* = 0.007, Fig. 2B), while originality was not (*r*_(11)_ = −0.44, *P* = 0.11, Fig. 2C).

We first examined the effects of stimulation, and stimulation site (DN node compared to loci outside of the DN) on creative thinking. A two-way ANOVA was conducted to examine the effects of DCS and Stimulation Site on the AUT Fluency Score. This analysis revealed an effect of DCS on Fluency, which was reduced following stimulation (*F*(1,11) = 7.47, *P* = 0.019), but none with Stimulation Site (*F*(1,11) = 0, *P* = 0.99). Surprisingly, no interaction was found between DCS and Stimulation Site (*F*(1,11) = 0, *P* = 0.72), suggesting a mismatch between preoperative planning and operative localization of the DN. A two-way ANOVA comparing the effects of DCS and Stimulation Site on Originality Score revealed no effects (Stimulation: *F*(1,11) = 0, *P* = 0.99; Stimulation Site: *F*(1,11) = 0.79, *P* = 0.39; Stimulation × Stimulation Site: *F*(1,11) = 1.79, *P* = 0.21). These findings indicate that in our cohort, DCS of the left hemisphere specifically affected creative fluency but not originality, and this effect is independent of planned stimulation site.

**Fig. 3:**
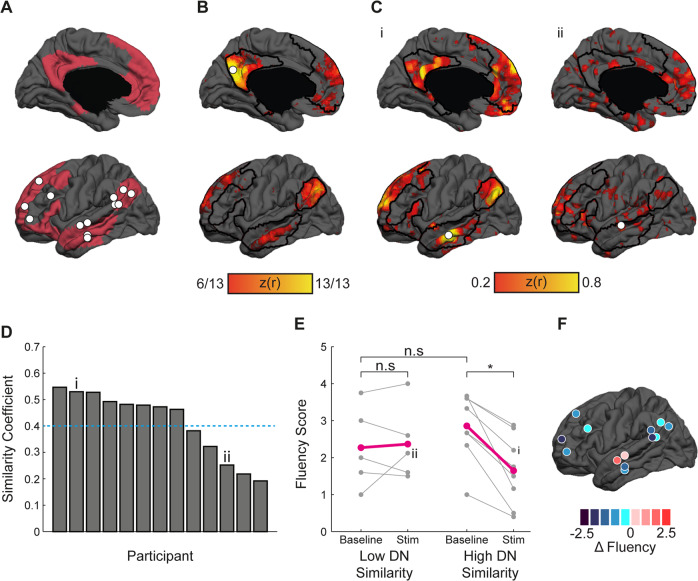
Direct cortical stimulation of the DN impairs creative fluency. **A** DN spatial extent adopted from Yeo et al. [3] is depicted. Direct cortical stimulation sites in the DN (*n* = 9) and in control regions (*n* = 4) are depicted (filled white circles). **B** The entire cohort (*n* = 13) DN map was extracted using a PCC seed region (denoted by a filled white circle). The map shows the convergence of individual PCC maps thresholded at *z*(*r*) of 0.2. **C** Representative individual participants’ maps derived from a seed region of interest within the DN placed at the stimulation site (filled white circle). A representative participant that demonstrates a map resembling the canonical DN (**i**), and a participant that does not (**ii**). **D** Individual participants overlap with the canonical DN (similarity coefficient) between the map obtained from the stimulation site ROI and the canonical DN. Participants i and ii are marked. **E** Participants with a similarity coefficient of 0.4 and above demonstrated significant impairment in creative fluency following direct cortical stimulation (*P < 0.05, n.s. non-significant; participants i and ii are marked, purple lines represent group average). **F** The relationship between direct cortical stimulation anatomical location and stimulation-induced changes in fluency are denoted as the difference in score change from baseline to the stimulation condition (filled dark-blue to dark-red circles).

Next, in order to further validate these findings and to minimize the potential effects of anatomical or surgically-associated disruptions on stimulation accuracy, such as intraoperative brain shift, we conducted a post-hoc analysis in which a seed was placed at the final point of stimulation (as was recorded intraoperatively using the navigation system). These ROIs (Fig. 3A) were used to calculate individualized whole-brain correlation maps, which were then compared to the canonical DN as defined by Yeo et al. [3]. First, we validated that the group DN map as derived from a PCC seed region overlaps with the canonical DN map (Fig. 3B). Next, we hypothesized that in participants where the final stimulation location derived seed-based maps are more similar to canonical DN, DCS will yield a more prominent effect on creative thinking (see Fig. 3C for individual participant’s examples of a stimulation ROI derived maps that show high (i) vs. low (ii) similarity to the canonical DN). Next, for each participant, we computed the overlap between the individualized stimulation-based map and the canonical DN map using the Sørensen-Dice similarity coefficient score (Fig. 3D). Then, we divided the cohort into two subgroups based on the Similarity score, and examined the effects of DCS in these two subgroups (Fig. 3E). A two-way ANOVA was conducted to examine the effect of Stimulation, and Similarity on the AUT Fluency Score. The analysis revealed that while Similarity did not have a direct effect on Fluency (*F*_(1,11)_ = 0.02, *P* = 0.91), it demonstrated a robust interaction with Stimulation (*F*_(1,11)_ = 12.62, *P* = 0.005), with a stronger effect of stimulation in individuals in the high Similarity subgroup. Importantly, examining this effect in individuals based on the anatomical location of the stimulation site (Fig. 3F), we found that the change in Fluency could be produced by stimulation of parietal, frontal and temporal regions, suggesting that it is network-dependent. In addition, no effect or interaction were found for Originality (Similarity: *F*_(1,11)_ = 0.1, *P* = 0.76; Stimulation ×Similarity: *F*_(1,11)_ = 0.56, *P* = 0.47). Collectively, these findings suggest that stimulation in areas that are more connected to the DN (as defined by the canonical DN) produces a strong effect on fluency, but not originality.

## Discussion

Using a novel experimental approach—disruption of individually identified regions within the default network using direct cortical stimulation in awake patients performing a divergent thinking task—we demonstrate a causal link between the default network and creative fluency. Stimulation of left (dominant) hemisphere DN nodes had no effect on originality scores, and stimulation outside the DN did not cause any disruption to creative thinking. We further validated this finding by demonstrating that direct cortical stimulation-induced impairment of creative fluency was specific to and more prominent in participants who were stimulated in DN-associated regions.

Creative cognition, a hallmark of higher cognitive function, is essential for human evolution and progress. The ability to perform creative thinking in both abstract forms, such as art, and in complex problem solving, such as science, necessitates multimodal information integration together with introspective thought processes. This manifests in the ability to link seemingly unrelated elements [25, 26]. These qualities may be explained by the unexpected synchronization of spatially segregated nodes of different computational modalities. Recent creative cognition models have postulated that creativity emerges through a synchronization between three cortical networks—the DN, salience and the executive control network [11, 27]. Each of these networks is considered to accommodate a different aspect of creative thinking. The DN, a network that mediates spontaneous cognition, or the “stream of consciousness”, is thought to contribute to the flexible retrieval of memories and generation of ideas [7]. The salience network is thought to filter useful and novel candidate ideas and forward them to the executive control network that constrains this stream towards a specific goal [28]. Various studies have correlated DN integrity or strength of internal correlation between different hubs with creativity psychometrics. Takeuchi et al. showed that reduced task-induced deactivation of the PCC is associated with increased creativity, postulating that reallocation of cognitive resources to DN domains instead of working memory may be responsible for enhanced creative thinking [29]. Abraham et al. used a conceptual expansion fMRI task, a specific form of divergent thinking, and found that both salience and DN nodes are overactivated in participants with higher creativity, suggesting that individual differences are dependent on network integration [30]. Benedek et al. [31], using a block design fMRI AUT, examined the functional organization of divergent thinking. In their study of creative idea generation, a deactivation of ventral attention components, and of the PCC within the DN, was observed, while increased activity was noted in other DN nodes, such as the prefrontal cortex, and the hippocampal formation. These results suggest that different aspects of creativity are modulated by specialized DN subcomponents. In another work, examining chains of free associations, Marron et al. demonstrated substantial involvement of the DN in free association production compared to other forms of language production [32]. Interestingly, in their study the majority of reported activations were in the left hemisphere, providing a possible explanation for our finding of decreased creative fluency following DN DCS. The emerging concept of sub-networks within the DN (for review see Buckner and DiNicola [33]) that are associated with various aspects of creativity is further supported by Shamay-Tsoory et al. [21]. This study, on which we relied methodologically, examined patients with localized lesions utilizing the same version of the AUT used here. Their finding of a correlation between right hemispheric lesions in the mPFC and decreased originality led them to associate this region with less linear cognitive processes. Interestingly, this finding is in line with our results demonstrating reduced creative fluency with DCS of left hemisphere DN and no change in originality, further suggesting a role for right hemisphere DN in original thinking. It should be noted that verbal and creative fluency are well correlated, and may partially overlap in function [34, 35]. However, in all participating subjects, the cortex exposed during surgery was thoroughly mapped for language functions (stimulated systematically while evaluating for naming, comprehension, and free speech). In all of the included subjects there was no disruption of speech-related functions, suggesting that the observed effect is creative fluency-specific, and not related to language disruption in general, further emphasizing the role of the DN in creative cognition. In addition, none of the patients were stimulated in the left inferior frontal gyrus, a main node in verbal fluency, which may disrupt both of these overlapping functions. In addition, recent evidence supports the existence of at least two DN subnetworks that are functionally specialized, and anatomically distinct in individual patients [36]. Two main subcomponents of the DN were recently characterized as mediating either theory of mind, or episodic projection [37]. This intra-DN parcellation may, in part, explain the variability in our participants’ performance, as well as the fact that only some components of creativity were decreased by DCS of an individual’s DN. An alternative explanation for the selective disruption of creative fluency seen following DN DCS is that the main contribution of the DN to divergent thinking is through mediation and retrieval of memories (for review see Hass and Beaty, 2018 [38]). This possible interference with episodic retrieval, is in line with recent evidence that boosting of episodic memory led to better performance in AUT [39]. As it is extremely rare for patients harboring gliomas in the non-dominant hemisphere to undergo awake resection, a complementary experiment adjudicating these two possible explanations (i.e., demonstrating reduced originality following right-sided DN DCS) was not performed in our particular setting, but is a subject for future research.

Several attempts were made over recent years to map, using electrical stimulation, what is termed “non-eloquent” cortex [40]. One such attempt by Foster et al. targeted the postero-medial cortex, a key node of the DN, via depth electrodes implanted in epilepsy patients [41]. In their study, and in concordance with our experience, when mapping areas of associative networks, no effect was evident. This lack of cognitive effects may be explained by the lack of an appropriate task or by the lack of patient-specific, network-based targets for stimulation. In our study design, the stimulated areas that yielded significant behavioral effects upon stimulation were those that produced a DN-like map at the individual level. Also, patients who demonstrated cognitive impairments or lack of creative ability in the preoperative neuropsychological assessment were excluded, as they were not expected to be able to fully participate in such a task.

We are aware of the limitations in our study design, as the number of subjects is limited and stimulation areas were limited to the left hemisphere. In addition, the number of task items and the time allowed for each item may influence the performance in AUT [42]. However the time frame in this special setting is extremely constrained [43], and we believe that this effect was minimized by the study design in which each participant served as their own control with no comparison of creative ability to an external cohort.

These limitations are a derivative of the unique experimental setting in which the human cortex is stimulated while an awake patient performs a task in the operating room. Recently, Natu et al. demonstrated that stimulation of the PCC in epilepsy patients implanted with depth electrodes impairs episodic memory recall, while no other subjective experience was elicited. In addition, following extended periods of stimulation of the PCC, a modulation of the PCC-hippocampal network, a key component of the DN, was evident [44]. These works, taken together with the findings presented here, set the stage for future interventional studies targeting associative networks-related functions that may greatly advance our understanding of the complex processes that lie at the core of human essence. These findings suggest a future approach for the mapping and preservation of creativity and higher cognitive functions in patients undergoing brain surgery.

## Supplementary information

Supplementary table 1
