# Supplementary material for: The default network is causally linked to creative thinking
Source: Mol Psychiatry. 2022 Jan 1;27(3):1848–54. doi: 10.1038/s41380-021-01403-8 (PMC9095481; doi:10.1038/s41380-021-01403-8)
Supplement: Supplementary file 1 — Supplementary table 1 [file 41380_2021_1403_MOESM1_ESM.docx]

**The default network is causally linked to creative thinking**

Ben Shofty^1,†, *^, Tal Gonen^1,2†^, Eyal Bergmann^3^, Naama Mayseless^4,5^, Akiva Korn^1^, Simone Shamay-Tsoory^4^, Rachel Grossman^1^, Itamar Jalon^2,5^, Itamar Kahn^3,‡,**^, and Zvi Ram^1,‡^

Supplementary table 1: preoperative global cognitive score as evaluated using NeuroTrax computerized cognitive testing battery (NeuroTrax Corp., Bellaire, TX).

| Patient number | Global Cognitive Score | Memory | Executive Function | Visual Spatial | Verbal Function | Attention |
| --- | --- | --- | --- | --- | --- | --- |
| 1 | 88.8 | 89.8 | 69.6 | 114.3 | 103.6 | 66.8 |
| 2 | 102.1 | 106.4 | 96.7 | 119.1 | 103.6 | 84.5 |
| 3 | n/a | n/a | n/a | n/a | n/a | n/a |
| 4 | 101.2 | 81.6 | 104.1 | 111.6 | 111.2 | 97.7 |
| 5 | 93.0 | 77.1 | 81.4 | 120.0 | 103.5 | 83.2 |
| 6 | 98.4 | 103.0 | 103.0 | 95.2 | 96.0 | 95.0 |
| 7 | 102.9 | 75.0 | 118.7 | 106.4 | 101.0 | 113.2 |
| 8 | 107.1 | 101.4 | 104.1 | 110.2 | 110.4 | 109.3 |
| 9 | 99.9 | 96.1 | 89.4 | 120.5 | 109.3 | 84.0 |
| 10 | 109.5 | 110.1 | 102.1 | 128.7 | 110.1 | 96.4 |
| 11 | 81.0 | 60.8 | 74.4 | 94.4 | 100.9 | 74.7 |
| 12 | 98.1 | 90.2 | 102.2 | 105.0 | 102.3 | 91.1 |
| 13 | 104.8 | 103.4 | 110.0 | 95.2 | 103.5 | 112.0 |

Normalized scores are standardized relative to cognitively healthy individuals of similar age and educational level and fit to a scale with mean=100 and SD=15.
